# Identifying the Potential Role and Prognostic Value of the Platelet-Derived Growth Factor Pathway in Kidney Renal Clear Cell Carcinoma

**DOI:** 10.1155/2022/9498010

**Published:** 2022-03-17

**Authors:** Shiyong Xin, Xianchao Sun, Liang Jin, Zhen Zhou, Xiang Liu, Weiyi Li, Wangli Mei, Jiaxin Zhang, Bihui Zhang, Xudong Yao, Liqing Zhou, Lin Ye

**Affiliations:** ^1^Department of Urology, Shanghai East Hospital, School of Medicine, Tongji University, Shanghai 200072, China; ^2^Department of Urology, Shanghai Tenth People's Hospital, School of Medicine, Tongji University, Shanghai 200072, China; ^3^Department of Rheumatology and Immunology, The First Affiliated Hospital and College of Clinical Medicine of Henan University of Science and Technology, Luoyang 471003, China

## Abstract

The platelet-derived growth factor (PDGF) pathway is important in angiogenesis, which can accelerate the formation of vessels in tumor tissues and promote the progression of malignant tumors. To clarify the role of PDGF in the occurrence of renal cell carcinoma and targeted drug resistance, we explored the pathway in kidney renal clear cell carcinoma (KIRC) through bioinformatics analysis with the aim of supporting comprehensive and individualized therapy. First, we found 40 genes related to the PDGF pathway through gene set enrichment analysis and then obtained their expressions and clinical data in 32 different cancers from The Cancer Genome Atlas (TCGA). Mutations in these genes (including copy number and single-nucleotide variation) and mRNA expression were also detected. Next, we conducted a hazard ratio analysis to determine whether the PDGF pathway genes were risk or protective factors in tumors. Although PDGF-related genes acted as traditional oncogenes and were closely related to tumor angiogenesis in many cancers, our results indicated that most genes had a protective role in KIRC. We further analyzed the methylation modification of PDGF pathway genes and found that they were prevalent in 32 different cancers. Furthermore, 539 KIRC samples obtained from TCGA were divided into three clusters based on the mRNA expression of PDGF genes, including normal, inactive, and active PDGF gene expressions. The results from survival curve analysis indicated that the active PDGF cluster of patients had the best survival rate. Using the three clusters, we studied the correlation between the PDGF pathway and 12 common targeted drugs, as well as classical oncogenes and infiltrating immune cells. A prognostic risk model was constructed based on the PDGF score using LASSO-Cox regression analysis to analyze the value of the model in predicting the prognosis of patients with KIRC. Finally, 11 genes were selected for LASSO regression analysis, and the results demonstrated the high predictive value of this risk model and its close relationship with the pathological characteristics of KIRC (metastasis, size, grade, stage, etc.). In addition, we found that the risk score was an independent risk factor correlated with overall survival through univariate and multivariate analyses and a nomogram was built to assess patient prognosis. In conclusion, the occurrence and development of KIRC may be associated with an abnormally activated PDGF pathway, which may be a potential drug target in the treatment of KIRC.

## 1. Introduction

As the most common renal cancer, renal cell carcinoma (RCC) accounts for about 3% of all adult malignant tumors and its incidence ranks third in urogenital malignant tumors [1]. RCC originates from proximal tubular epithelial cells, and kidney renal clear cell carcinoma (KIRC) is the most common pathologic type [2, 3]. There is no specific screening test for the RCC and 25%–30% of patients are already diagnosed with metastatic RCC or advanced renal RCC (aRCC) [4]. In addition, 20%–30% of patients have recurrence or metastasis after radical nephrectomy [5]. Since RCC is naturally insensitive to radiotherapy and chemotherapy, cytokine therapy based on interleukin-2 (IL-2) and interferon-*α* has been the standard treatment since 1990, but the objective response rate of cytokines was only 5%–27%, and the median progression-free survival (PFS) was only 3–5 months, with significant adverse reactions [6]. In the past decade, targeted drug therapy has been the best treatment for advanced renal cell cancer. In 2006, the prognosis of aRCC was significantly improved under the advent of molecular-targeted drugs, and the PFS of patients was twice that of those who received cytokine therapy, and overall survival (OS) was extended from 8 to 28–29 months [7]. The molecularly targeted drugs approved by the Food and Drug Administration for the treatment of aRCC mainly include sunitinib, sorafenib, pazopanib, axitinib, bevacizumab, everolimus, and temsirolimus. The first four are tyrosine kinase inhibitors, bevacizumab is a human monoclonal antibody, and the last two are mammalian targets of rapamycin (mTOR) inhibitors. The emergence of molecularly targeted drugs has changed the treatment of aRCC and improved the survival of RCC patients. Of note, although the effects of targeted drugs are better than cytokines, some patients are still found to have no or a poor response to molecularly targeted therapy in clinical practice. Moreover, the cause and mechanism of this phenomenon remain unclear, especially the mechanism of drug resistance. Consequently, it is urgent to further investigate the mechanism of drug resistance, deepen the understanding of the occurrence and development of RCC from the molecular level, and develop new targeted drugs.

As a member of the vascular endothelial growth factor (VEGF) family, PDGF is a polypeptide growth factor containing a glycochain with a relative molecular weight of about 30000 kD. PDGF is basically a homologous or heterologous dimer molecule formed by four polypeptide chains A, B, C, and D through disulfide bonds and has five subtypes PDGF-AA, PDGF-BB, PDGF-AB, PDGF-CC, and PDGF-DD [8, 9]. PDGF plays a biological role by binding the platelet-derived growth factor receptor-*α* (PDGFR-*α*) and PDGFR-*β* to activate the PDGFR pathway. PDGF-BB is the only ligand that can simultaneously activate PDGFR-*α* and PDGFR-*β*, indicating that PDGF-BB has a stronger biological effect in the activation of the PDGFR pathway [10–12]. Studies have shown that PDGF-BB is closely related to tumor growth, invasion, and migration, and the expression of PDGF-BB will enable cancer cells to recruit stromal and endothelial cells into tumors to promote their growth and development [13, 14]. The progressive growth and metastasis of tumors also depend on vascular development (angiogenesis) and the expression of PDGF-BB can promote the formation of tumor vessels. In recent years, angiogenesis has been considered to play an important role in the genesis, development, invasion, and metastasis of tumors. As an angiogenic factor, PDGF is closely related to the development of tumors [15]. The proliferation and migration of vascular endothelial cells and tumor cells could be induced by the PDGF that is released by tumor cells and plays a direct role in tumor angiogenesis. Overexpression of PDGF and its receptor is one of the common features of tumors. High levels of PDGF and receptors can be detected in breast, cervical, endometrial, skin, gastric, colorectal, lung, pancreatic, prostate, ovarian, and other tumor cells. Breast cancer cells can produce PDGF, stimulate the proliferation of adjacent fibroblasts with PDGF receptor, cause the formation of connective tissue within the tumor, and support the growth, invasion, and metastasis of tumor cells, which is also one of the common features of breast cancer [16–18]. PDGF promotes tumor genesis and development through autocrine and paracrine signaling mechanisms. The excessive expression of PDGF and its receptor through autocrine can lead to the occurrence of osteosarcoma, lung cancer, glioma, and medulloblastoma. Human sarcoma and glioma can secrete PDGF abnormally, acting on tumor cells and resulting in abnormal hyperplasia. PDGF autocrine signaling not only stimulates the proliferation of tumor cells but also promotes the invasion and metastasis of tumor cells in some epithelial cell types [19–22]. With the development of related studies, the role of PDGF/PDGFR in angiogenesis will become clearer, and blocking the PDGFR signal transduction of tumor cells may provide a new therapeutic target.

The mechanism of the PDGF pathway and its related genes in the occurrence and development of KIRC and its role in resistance to molecularly targeted drug therapy is still unclear. Therefore, detecting the relationship between the PDGF pathway and KIRC will be particularly meaningful. We first investigated the expressions of 40 genes related to the PDGF pathway and their mutation through gene set enrichment analysis (GSEA) and The Cancer Genome Atlas (TCGA). Next, we conducted a hazard ratio (HR) analysis to determine whether the PDGF pathway genes were risk factors or protective factors in tumors. We also analyzed the methylation modification of the PDGF pathway genes and its relationship with copy number variation (CNV) and mRNA expression in 32 different cancers. Subsequently, 539 KIRC samples obtained from TCGA database were divided into three clusters according to the mRNA expressions of PDGF genes: cluster 1, normal PDGF; cluster 2, inactive PDGF; and cluster 3, active PDGF. Then, based on the three clusters, we studied the correlation between the PDGF pathway and 12 common targeted drugs, as well as classical oncogenes and infiltrating immune cells. Finally, a prognostic risk model was constructed to analyze the value of the PDGF pathway in predicting the prognosis of patients with KIRC. Our study aims to clarify the role and related mechanism of the PDGF pathway in RCC and provide a theoretical basis for the comprehensive treatment and individualized treatment of RCC, especially KIRC.

## 2. Methods

### 2.1. Data Acquisition and Mutation Analysis of PDGF-Related Genes

We obtained 40 genes related to the PDGF pathway from the GSEA package (https://www.gseamsigdb.org/gsea/index.jsp), and their expressions, mutations (CNV and single-nucleotide variation (SNV)), and clinical data in 32 different cancers were from TCGA database (https://portal.gdc.cancer.gov). We analyzed the data with Perl language and *R* Studio and used Toolbox for Biologists (TBtools) to present the results [23]. For KIRC, we obtained the clinical and PDGF pathway gene expression data of 539 renal cancer patients from TCGA for further research. Furthermore, we used “corrgram” in RStudio (MA, USA) to show the expression relationship among PDGF pathway genes, and Pearson's correlation coefficient analysis was applied to analyze the results. With the development of epigenetic modification, DNA methylation has been found to play a significant role in kidney cancer. Therefore, we investigated the methylation difference in each cancer and its relation with CNVs and mRNA expression in KIRC.

### 2.2. Cluster Analysis of the PDGF Pathway Genes

Because of the large variation in gene expression, we constructed a PDGF scoring model to investigate the expression differences between samples we obtained from TCGA. First, we used a single-sample GSEA (ssGSEA) to calculate the enrichment scores of the PDGF pathway genes. Second, we divided the KIRC samples into three clusters by comparing the mRNA expressions in normal tissues: cluster 1 (C1), PDGF normal; cluster 2 (C2), PDGF inactive; and cluster 3 (C3), PDGF active. “gplots” in RStudio was used for differential analysis, and “pheatmap” was used to draw the heat map. Furthermore, the violin plot was drawn with “ggpubr” to indicate the enrichment score of three clusters. Finally, survival curves were plotted with “survival” in RStudio. *P* < 0.05 indicates statistical difference.

### 2.3. Correlation between the PDGF Pathway Genes and Drug Sensitivity

To investigate the effect of the PDGF pathway genes on molecularly targeted therapy of RCC and their relationship, 12 classic targeted drugs were selected from the Genomics of Drug Sensitivity in Cancer (GDSC) database (https://www.cancerrxgene.org/) for further analysis. The half-maximal inhibitory concentration (IC_50_) of the molecularly targeted drugs in the three clusters was calculated through a ridge regression model constructed with the “pRRopheticl” package according to the cell expression profiles in the GDSC database [24]. All parameters except for the “combat” and “allSoldTumours” tissue types were set to default values, and the levels of duplicate gene expression were aggregated as an average. The boxplot was drawn with “ggplot2” and “cowplot” packages. Ultimately, we analyzed the correlation between mRNA expression and drug sensitivity from GDSC and the Cancer Therapeutics Response Portal (CTRP), respectively. *P* < 0.05 indicates statistical difference.

### 2.4. Correlation between PDGF Pathway Genes and Classic Cancer-Related Genes

Hyperactivation and abnormal expression of PDGF can directly or indirectly promote the proliferation and migration of tumor cells. To clarify the mechanism of the PDGF pathway in KIRC, we further investigated the relationship between PDGF pathway genes and classic tumor-related genes. The “string,” “pheatmap,” “gplots,” and “gird” packages in RStudio were used to draw a heat map to determine the trend in expression of classic tumor-related genes in three PDGF clusters. One-way ANOVA was used to perform statistical analysis. It is well known that posttranscriptional histone modification plays a key role in the development of cancer, and histone modification has also been explored as a possible marker of disease occurrence and development. Therefore, we examined the difference in expressions of sirtuins (SIRTs) and histone deacetylases (HDACs) among the three PDGF clusters. *P* < 0.05 indicates a statistical difference.

### 2.5. Analysis of the Relationship between the PDGF Pathway and Immune Infiltrating Cells in KIRC

The distribution pattern of immune cells in the tumor microenvironment is closely related to the survival prognosis of patients, and the infiltrating pattern of immune cells can be used as the basis for treatment to improve the survival rate and long-term prognosis of patients [25]. At present, there are few studies on immune cell infiltration and immune markers in KIRC, and there is still a lack of accurate screening methods for the immunotherapy markers of renal cancer. In our study, 29 immune-related gene sets that could represent different immune cell types from TCGA were identified through ssGSEA for further study [26–28]. A heat map was drawn with “ggplot2” and “dplyr” packages in RStudio to indicate the relationship between the PDGF genes and immune cell infiltration in KIRC. Spearman's correlation was used in the statistical analysis. Furthermore, the “ggstatsplot,” “data.table,” “dplyr,” “tidyr,” and “ggplot2” packages in RStudio were used to analyze and show the relationship between the PDGF score and immune substances. Finally, the “ggscatterstats” package was used to draw a scatter diagram to demonstrate the relationship between the two classic immune cell groups and the PDGF score. *P* < 0.05 indicates a statistical difference.

### 2.6. Establishment of a Risk Model and Its Value in the Evaluation of Prognostic Prediction

First, we used “pheatmap” to draw a heat map and show the expression levels of PDGF pathway genes in normal and KIRC tissues. Then, the “corrplot” package was used to draw a heat diagram to demonstrate the coexpression relationship between any two genes in the PDGF pathway. Meanwhile, LASSO-Cox analysis was conducted to establish a risk model with the “glmnet” package in RStudio. Subsequently, the risk score (RS) per sample was computed through the formula: RS=∑_*i*=1_^*n*^coef_*i*_ × *x*_*i*_, where coef_*i*_ represents the coefficient and *x*_*i*_ represents the expression value of each selected gene. The samples were classified into a high-risk group and a low-risk group according to the best cut-off value obtained with the “survminer” package, and the “survival” package in *R* was used to obtain the survival curves of these two groups. In addition, we performed the “survival-ROC” package in *R* to draw the receiver operating characteristic (ROC) curve and calculate the area under the ROC curve. Moreover, the area under the curve value of each model was computed with “timeROC” package in *R*. Ultimately, a heat diagram was drawn to show the relationship between the RS and the clinicopathological characteristics of patients with KIRC. *P* < 0.05 indicates a statistical difference.

### 2.7. Validation of the Model with Nomogram

We performed univariate and multivariate Cox regression analyses to clarify the relationship between the clinicopathological characteristics of the KIRC patients and the RS. Ultimately, the “rms” package in RStudio was used to plot a nomogram for evaluating the prognosis of patients with KIRC.

## 3. Results

### 3.1. Mutation of PDGF-Related Genes in 32 Types of Cancers

**Figure 1:**
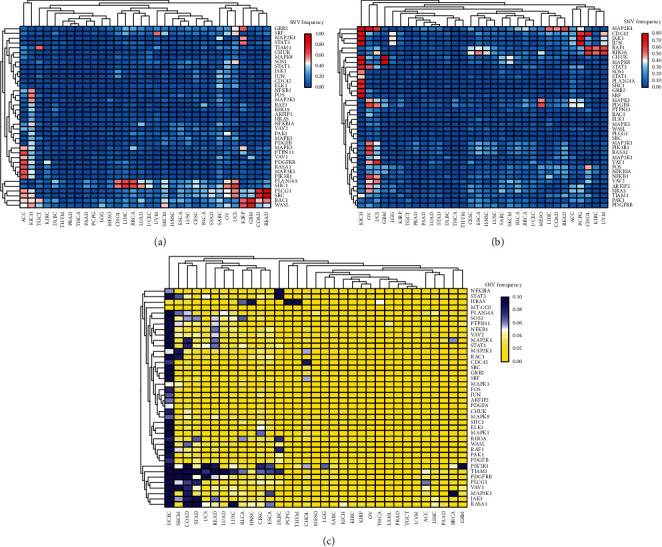
((a) and (b)) CNV frequencies of 40 PDGF pathway genes in 32 cancers from TCGA. The color bar: gain or lose copy number. (c) SNV frequencies of 40 PDGF pathway genes in 32 cancers from TCGA. The color bar: the degree of SNV (pink: high frequency, blue: low frequency).

Genetic heterogeneity between and within tumors is a major factor determining cancer progression and treatment response, and there are significant differences among different tumors and even within the same tumor. SNV and DNA CNV can also be manifested as interpatient heterogeneity (interpatient), intertumor heterogeneity (intertumor), and intratumor heterogeneity (intratumor). In our study, the CNV and SNV of 40 PDGF pathway genes in 32 tumors were studied through TCGA pan-cancer project. As can be seen from Figure 1, CNV gain and CNV loss existed in most tumors, except for THYM, PRAD, and THCA, and CNV gain of most PDGF pathway genes occurred in ACC and KICH, SARC, OV, and UCS. Moreover, CNV loss in most of the PDGF genes occurred in KICH, OV, and UCS (Figures 1(a) and 1(b)). Except for PRAD, TGCT, and UVM, SNVs in PDGF pathway genes existed in most tumors. SNVs fluctuated between 0.2 and 0.6 in most tumors except for UCEC, SKCM, COAD, STAD, and UCS (Figure 1(c)). Of note, only a few PDGF genes had CNV gain and loss in KIRC, and CNVs fluctuated between 0.2 and 0.6. As with CNV, the SNV of PDGF genes had a low incidence rate in KIRC, and SNVs fluctuated between 0.02 and 0.06 (Figures 1(a)–1(c)).

### 3.2. mRNA Expression and Methylation of PDGF Pathway Genes in Cancer

**Figure 2:**
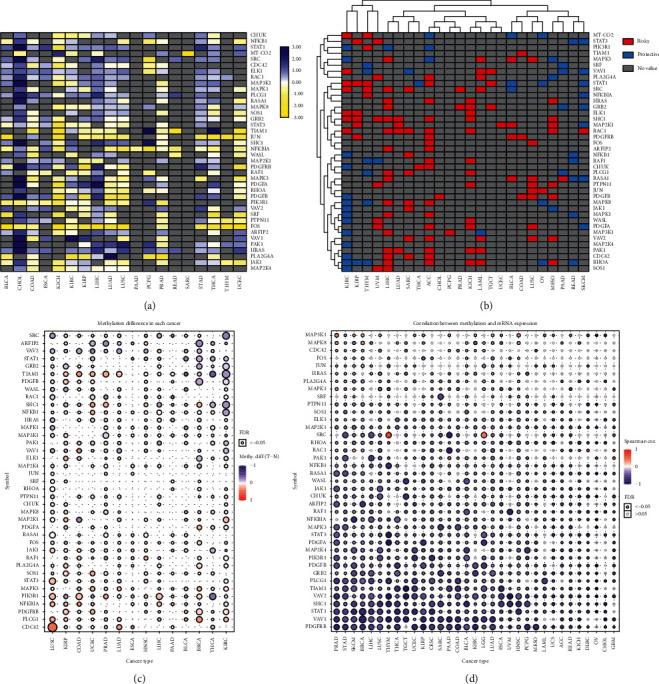
(a) Expressional changes of 40 PDGF pathway genes. The color code bar: corresponding value of log2 (FC). (b) The role of different PDGF genes for different cancers. Pink: risky gene, blue: protective gene, and gray: no statistical significance. (c) Methylation difference in 14 different types of cancer; the color bar means difference. (d). Correlation between methylation and mRNA expression in 32 cancers. The color bar means correlation coefficient.

The mRNA expression of the PDGF-related genes in 19 types of cancers was investigated (Figure 2(a)). Our results showed that the mRNA expression level of most PDGF-related genes in cancer was upregulated except for PAAD (Figure 2(a)). Next, the genes involved in the PDGF pathway had protective or risk roles, which was determined from the relationship between patients survival rates and their expression level (Figure 2(b)). The expression of a protective gene provides a better prognosis when upregulated, whereas the upregulated expression of a risk gene has a decreased survival rate, which was decided in our study based on HR (<1: protective and >1: risk). Studies have demonstrated that PDGF closely correlates with carcinogenesis, growth, invasion, and migration. Furthermore, PDGF enables cancer cells to recruit stromal cells and endothelial cells into tumors, which promotes their growth and development [13, 14]. Because of its role as a tumor promoter, genes related to the PDGF pathway have risk roles in most cancers (Figure 2(b)). However, our results found that most PDGF-related genes played a protective role in KIRC, which contradicted previous results (Figures 2(a) and 2(b)). Furthermore, to detect the mechanism of PDGF genes in tumors, we analyzed the methylation of PDGF-related genes in 14 common cancers and analyzed the relationship between methylation and mRNA expression. In addition, we analyzed the relationship between the methylation of PDGF-related genes and CNV and patient prognosis by GSCA (https://bioinfo.life.hust.edu.cn/GSCA/#/). We found widespread methylation differences in PDGF-related genes occurred in 14 cancers compared with normal tissue. The results also showed a negative correlation between the methylation of PDGF-related genes and mRNA expression in 32 types of cancers from TCGA, which indicated that the methylation of PDGF-related genes may act before the transcription and play a significant role in carcinogenesis and progression (Figures 2(c) and 2(d)). As with other tumors, the methylation of PDGF-related genes was negatively correlated with mRNA expression in KIRC, especially VAV1, PDGFRB, VAV2, PDGFB, and GRB2.

### 3.3. The Methylation, CNV, and mRNA Expression of PDGF Genes Related to the Survival of KIRC Patients

**Figure 3:**
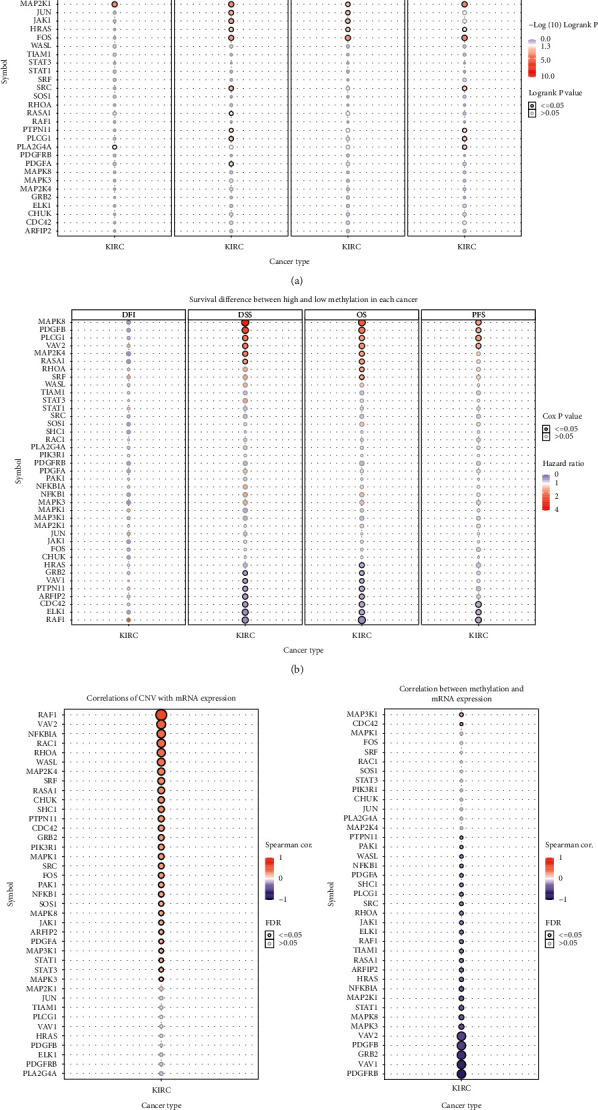
(a) Survival difference between CNV groups in KIRC. (b) Survival difference between high methylation and low methylation in KIRC. (c) Correlations of CNV with mRNA expression in KIRC. (d) Correlation between methylation and mRNA expression in KIRC.

Gene methylation and CNV can affect the stability of gene expression, thus affecting gene transcription and expression, which is closely related to the occurrence of many diseases, especially in malignant tumors. As an important cancer-promoting factor, CNV and methylation of genes related to the PDGF pathway are widely present in cancer, and methylation of the PDGF genes is negatively correlated with mRNA expression in most tumors according to our results. To further clarify the role of CNV and methylation in PDGF-related genes in KIRC, we investigated the relationship between the methylation of PDGF-related genes and the prognosis of patients with KIRC through GSCA (https://bioinfo.life.hust.edu.cn/GSCA/#/). First, we investigated the difference of disease-free interval (DFI), disease-specific survival (DSS), OS, and PFS of KIRC patients in different CNV groups by using the log-rank test. As shown in Figure 3(a), DSS, OS, and PFS were significantly different in CNV groups, suggesting that the CNV of the PDGF genes could significantly affect the prognosis of KIRC patients (Figure 3(a)). Next, the Cox regression test was used to analyze the differences in the DFI, DSS, OS, and PFS in KIRC patients between high and low methylation PDGF genes. The results are shown in Figure 3(b); similar to the CNV, there were significant differences in DSS, OS, and PFS between the high and low methylation groups, suggesting that the methylation of these PDGF genes could significantly affect the prognosis of KIRC patients (Figure 3(b)). Finally, Pearson correlation analysis was used to detect the correlation between CNV and mRNA expression in PDGF-related genes, as well as between methylation and mRNA expression of PDGF-related genes in KIRC. The results showed a positive correlation between CNV and mRNA expression, while a negative correlation existed between methylation and mRNA expression in most PDGF-related genes (Figures 3(c) and 3(d)).

### 3.4. Cluster Classification and Analysis Based on the PDGF Scores

**Figure 4:**
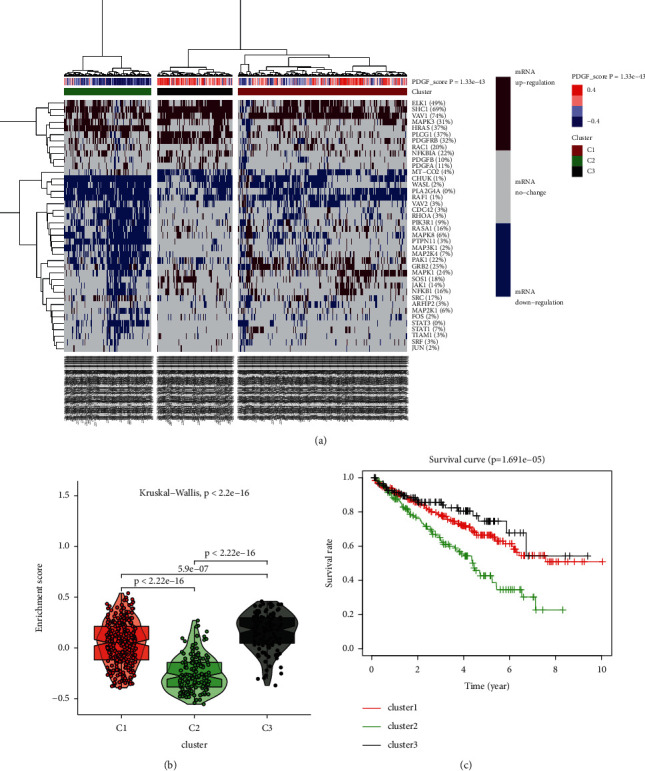
(a) KIRC samples were divided into 3 PDGF clusters. (b) Violin plot shows the enrichment score of 3 PDGF clusters. (c) Survival curves of 3 clusters.

Consistent with previous studies, our results suggest that PDGF pathway genes play a carcinogenic role in most cancers. However, in KIRC, most PDGF pathway genes are protective. Therefore, it is necessary to further study the mechanism of the PDGF pathway in KIRC. To facilitate subsequent research, we constructed a PDGF scoring model based on the expression of 40 PDGF genes (Figure 4(a)). Subsequently, 539 KIRC samples were divided into three clusters according to the PDGF score: cluster 1, normal PDGF; cluster 2, inactive PDGF; and cluster 3, active PDGF. Next, we drew a violin diagram to show the order of the PDGFP scoring of the three clusters: C3 > C1 > C2 (Figure 4(b)). Finally, we plotted the survival curves and showed that the PDGF active group (C3) had the best survival rate (Figure 4(c)), which was consistent with the previous results, suggesting that PDGF plays a protective role in KIRC.

### 3.5. Relationship between the PDGF Pathway and Classical Genes and Histone Modification in KIRC

**Figure 5:**
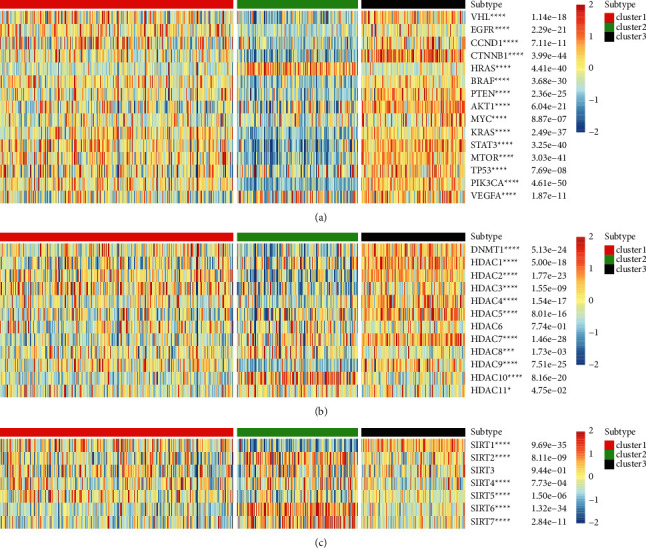
(a) The correlation between PDGF genes and classical genes. (b) The correlation between PDGF genes and HDAC family genes. (c) The correlation between PDGF genes and sirtuin family genes.

First, we investigated the expressions of classic cancer-related genes in three clusters. Through analysis of the results, we could understand the expression relationship between these genes and PDGF genes in KIRC. As shown in the heat map of Figure 5(a), there was a significant difference among the three clusters in the expressions of tumor-related genes (Figure 5(a)). Furthermore, consistent with the expression trend of PDGF genes in the three clusters, tumor-related oncogenes were remarkably upregulated in the PDGF active group (C3) and downregulated in the PDGF inactive group (C2) except for HRAS. Interestingly, the expression of HRAS in the PDGF inactive group (C2) was significantly higher than that in the PDGF active group (C3), which was the complete opposite to other oncogenes. We found that the expression of tumor suppressor genes was significantly higher in C3 than in C2. However, it was noteworthy that trends in the expressions of Von Hippel-Lindau (VHL), TP53, and PTEN were consistent with the oncogenes (Figure 5(a)). The above results demonstrated that the poor prognosis of the PDGF inactive group (C2) may result from the inhibition of the tumor suppressor gene and the upregulated expression of the oncogene HRAS.

With further studies on the genetic factors of renal cancer, epigenetic modification was found to have an important role in the development of tumors. Among the epigenetic modification processed in tumors, histone modification plays a key role. Epigenetic mechanisms mediated by HDACs, histone acetyltransferases, and HMTs play key roles in cell proliferation, angiogenesis, hypoxia-related effects, and cell cycle regulation. Histone deacetylases, which remove acetyl groups from histone and nonhistone lysine residues, play an important role in the regulation of gene transcription and are implicated in the development and metastasis of a variety of tumors. Recently, the inhibition of HDAC has emerged as a clinically proven cancer treatment strategy [29, 30]. In our study, apart from HDAC6, we found significant differences in the expression of HDCAs in the three clusters. As shown in Figure 5(b), HDAC1, HDAC5, HDAC7, HDAC8, HDAC10, and HDAC11 expressions were significantly higher in C2 than in C1, indicating that they may be associated with poor prognosis of C2 (Figure 5(b)). Currently, our results may further support the inhibition of HDACs in the treatment of RCC. For instance, HDAC10 was significantly upregulated in the inactive group but was normal in the active group. Therefore, treatment with an HDAC10 inhibitor may be more beneficial to KIRC patients with the inactivation of genes in the PDGF pathway.

Currently, SIRT is known to be involved in a variety of cancer-related molecular biological processes such as tumor-related metabolism, changes in the tumor microenvironment, and abnormal cell proliferation. Moreover, SIRT works as an oncogene or tumor suppressor [31]. Our results showed that there were significant differences among the three clusters on the expressions of other SIRTs except for SIRT3. As shown in Figure 5(c), the expressions of SIRT1 and SIRT5 in the PDGF inactive group (C2) were significantly lower than those in the PDGF active group (C3), while those of SIRT2, SIRT4, SIRT6, and SIRT7 in the PDGF inactive group (C2) were significantly higher than those in the PDGF active group (C3) (Figure 5(c)). Li et al. found that the ethanol extract of *Patrinia scabiosifolia* induces the death of human RCC 786-O cells via SIRT-1 and mTOR signaling-mediated metabolic disruptions [32]. In summary, these results suggested that SIRT2 inhibitors may be more effective in patients who belong to the PDGF-inactive group (C2).

### 3.6. Relationship between Classic Molecularly Targeted Medicine and PDGF

**Figure 6:**
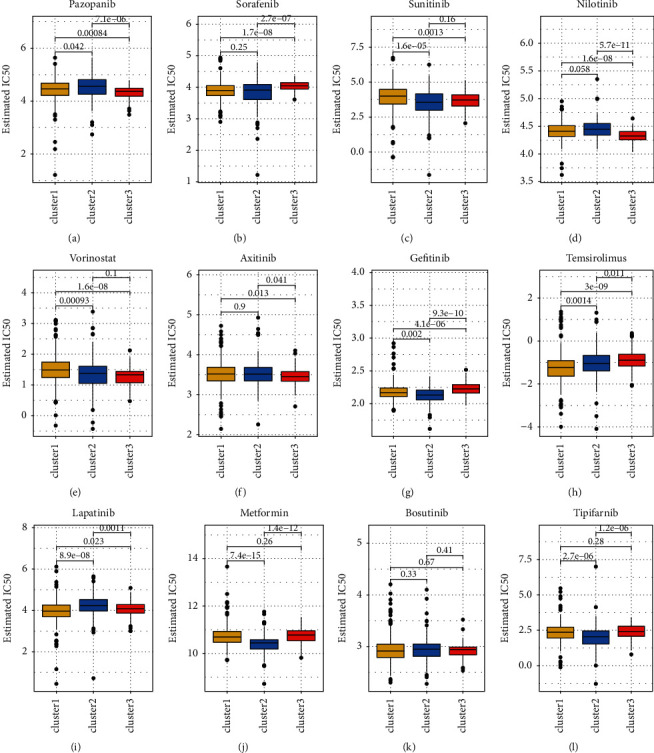
The estimated IC_50_ for 12 types of common chemotherapeutic agents are shown in the plot for 3 PDGF clusters.

**Figure 7:**
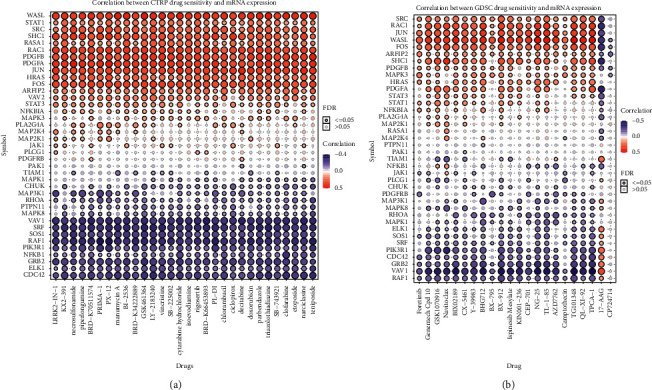
(a) Correlation between CTRP drug sensitivity and mRNA expression of PDGF pathway genes. (b) Correlation between GDSC drug sensitivity and mRNA expression of PDGF pathway genes.

To clarify the correlation between the PDGF pathway and molecularly targeted therapy for advanced RCC, a drug sensitivity analysis was performed with GDSC to investigate in depth the drug sensitivity among the three PDGF clusters. At present, there are many kinds of molecularly targeted drugs in clinical use, and their mechanisms of action are also different. For example, imatinib is a tyrosine kinase inhibitor and PDGFR kinase inhibitor, which blocks the activity of oncoprotein BCR-ABL and cell surface receptor tyrosine kinase C-Kit. Sorafenib is an oral multitarget multikinase inhibitor that can target serine/threonine kinases and receptor tyrosine kinases on tumor cells and tumor blood vessels and has the dual effect of simultaneously inhibiting tumor cell proliferation and angiogenesis [33]. Sunitinib is a multitarget tyrosine kinase inhibitor targeting PDGFR, VEGFR, C-kit, and FLT-3 with dual antitumor and antiangiogenic effects [33]. Gefitinib is an EGFR inhibitor [34], bosutinib inhibits the tyrosine kinase inhibitor (TK) [35], and axitinib is a VEGF pathway inhibitor [36]. Metformin is an antidiabetic drug that can reduce glycemia to block the PI3K/MAPK pathway [37]. In the past decade during clinical work, we have found that some patients with advanced RCC do not respond well to targeted drug therapy, which shows the importance of detecting the correlation between these targeted drugs and the PDGF pathway. A drug sensitivity analysis was performed to estimate the IC_50_ values of the drugs for each sample. A lower IC_50_ always indicates better drug efficacy. A ridge regression model was conducted to reveal the drug sensitivities among the three clusters as follows (Figure 6): pazopanib-C3 > C1 > C2; sorafenib-C1 > C3, C2 > C3; sunitinib-C2 > C1, C3 > C1; nilotinib-C3 > C1, C3 > C2; vorinostat-C3 > C2 > C1; axitinib-C3 > C2, C3 > C1; gefitinib-C2 > C3 > C1; temsirolimus-C1 > C2 > C3; lapatinib-C3 > C1 > C2; metformin-C2 > C1 > C3; bosutinib-no difference; and tipifarnib-C1 > C2, C3 > C2. Based on the above results, we may have a better understanding of the effects of the drugs among the clusters, which will help to establish precise treatments for KIRC in the future. For example, if a patient with advanced KIRC has a high PDGF pathway gene expression and high PDGF score, we may obtain a good therapeutic result with vorinostat or pazopanib. Finally, the correlation between GDSC drug sensitivity and the mRNA expression of PDGF genes was investigated through the GDSC database. In addition, we further studied the correlation between drug sensitivity and mRNA expression using the CTRP website. As shown in Figures 7(a) and 7(b), the drug sensitivity data from GDSC and CTRP correlate closely with the mRNA expression of most PDFG pathway genes. However, some correlations were positive and some were negative (Figure 7). For example, because of the positive correlation, ARFIP2, FOS, HRAS, JUN, PDGFA, PDGFB, RAC1, RASA1, SHC1, SRC, STAT1, and WASL had higher mRNA expression that corresponded with higher drug sensitivity (Figure 7(a)).

### 3.7. Immune Cell Infiltration Correlated with PDGF Score in KIRC

**Figure 8:**
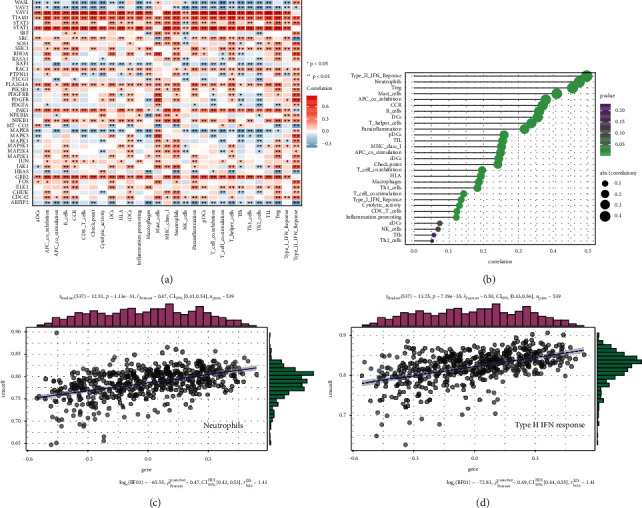
(a) Correlation between PDGF pathway and immune infiltrating related substances. Pink: positive and blue: negative. (b) Degree of correlation, the area of the sphere: the abs (correlation) and the color: *P* value. (c, d) The scatter plot about the specific relationship between two immune infiltration-related substances and PDGF score.

The immune model of the tumor microenvironment is one of the characteristics of tumors, which can not only predict the survival prognosis of patients but also predict the efficacy of radiotherapy and chemotherapy. The distribution pattern of immune cells in the tumor microenvironment closely correlates with the survival prognosis of patients, and the infiltrating pattern of immune cells can be used as the basis for treatment to improve the survival rate and long-term prognosis of patients [25]. As an indispensable part of the microenvironment, tumor-infiltrating immune cells correlate closely with tumor development and drug resistance and are effective targets for antitumor therapy [38]. Therefore, it is meaningful to investigate the relationship between the PDGF pathway and immune cell infiltration in KIRC, which was analyzed through conducting a correlation analysis. As shown in Figure 8(a), we found that all the PDGF pathway genes correlated with the infiltrating immune substance. Of note, STAT1, STAT3, TIAM1, VAV1, SRC, RAC1, PLA2G4A, PAK1, NFKB1, and GRB2 had a significantly positive association with immune infiltration cells. However, VAV2, WASL, RAF1, MAPK8, and ARFIP2 had a significantly negative association with the infiltration of immune cells (Figure 8(a)). Furthermore, for most infiltration immune cells, especially Treg, neutrophils, and Type II IFN response, we found a remarkable correlation with PDGF pathway genes and a significant difference, except for Th2 cells, Tfh, NK cells, and aDCs (Figure 8(b)). Subsequently, our results indicated that the PDGF score positively correlates with the infiltration of type II IFN response cells and neutrophils (Figures 8(c) and 8(d)).

### 3.8. Risk Model Constructed to Predict Prognosis of Patients with KIRC

**Figure 9:**
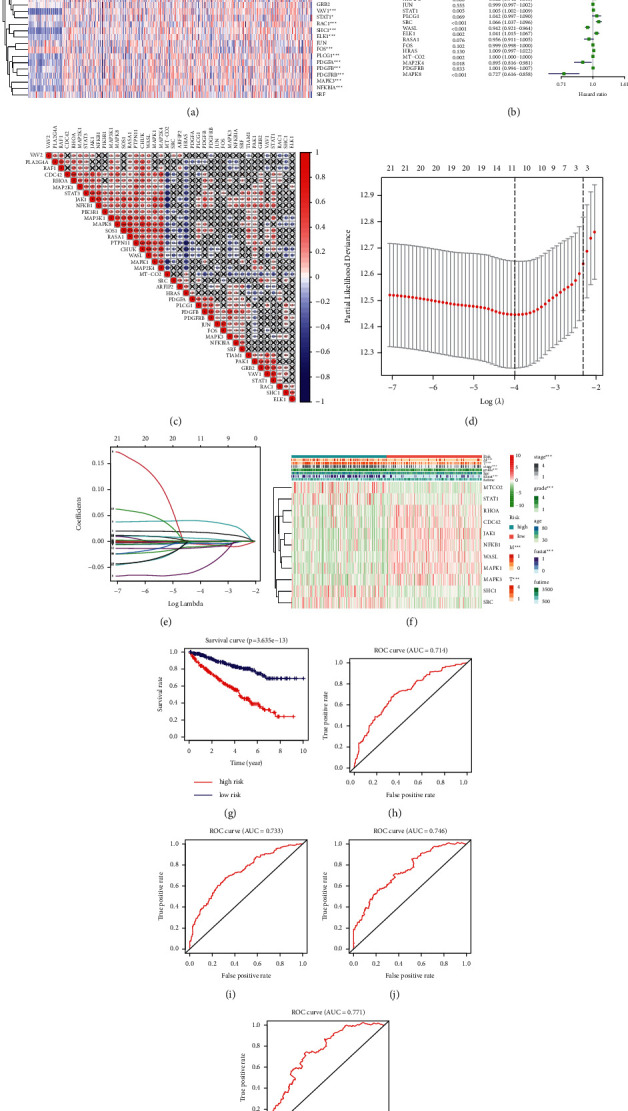
(a) The expression of 40 PDGF pathway genes in KIRC (pink: upregulation, blue: downregulation; N (blue): normal sample, T (red): tumor sample). (b) Hazard ratios (HR) analysis with 95% confidence intervals (CI) and *P* values. (c) Coexpression analysis of 40 PDGF pathway genes. (d) The LASSO coefficient profiles of PDGF pathway genes in KIRC. (e) 22 genes were selected by LASSO-Cox regression analysis. (f) The correlation of 11 finally selected genes and the clinicopathological characteristics in two groups (the color bar: the expression of genes, pink: upregulation, blue: downregulation). (g) The survival curve obtained based on PDGF risk model. Pink: high-risk group, blue: low-risk group. (h–k) ROC curve of 3, 5, 7, and 10 years.

First, we compared the expression of 40 PDGF pathway genes in 72 normal tissue samples with 539 KIRC tissue samples from TCGA. The results showed that 33 genes in the PDGF pathway were significantly differentially expressed, and the expressions of VAV2, PLA2G4A, RAF1, STAT3, PTPN11, CHUK, MAP3K1, MAPK8, CDC42, RHOA, MT-CO2, ARFIP2, and FOS were higher in the tumor group than in the normal group. However, the expressions of HRAS, VAV1, RAC1, SHC1, PLCG1, PDGFA, PDGFB, PDGFRB, MAPK3, and NFKBIA were lower in the tumor group than in the normal group (Figure 9(a)). Subsequently, an HR analysis was used to analyze the correlation between the PDGF pathway genes and the progression of KIRC (Figure 9(b)). We found that 25 genes in the PDGF pathway were associated with KIRC progression with a statistical difference (*P* < 0.05), among which 12 genes had a highly significant difference (*P* < 0.001). Next, we used a coexpression analysis to investigate the relationship between the 40 PDGF pathway genes (Figure 9(c)). Furthermore, a RS model was built to determine whether it could predict the outcomes of patients with KIRC. In addition, a LASSO-Cox regression analysis of the 22 genes was conducted, and 11 PDGF genes were finally used to construct the model (Figures 9(d) and 9(e)). Based on their RS values, we classified patients into high-risk and low-risk groups. A heat map was drawn to show the relationship between the risk model and the pathological features of KIRC, and a significant correlation was shown (Figure 9(f)). A survival curve was drawn to show the difference between the two groups. The results showed that the high-risk group had a lower OS rate than the low-risk group (Figure 9(g)). Finally, an ROC curve analysis was conducted to investigate the prognostic prediction efficiency of the new survival model. The results showed that the areas under the ROC curves of the survival model were 0.714, 0.733, 0.746, and 0.771, which illustrated that they had good predictive values (Figures 9(h)–9(k)). A higher predictive accuracy was shown by our prognostic model than with the signature constructed by Yu et al., which predicted that the survival time of patients with KIRC was 1 year and 4 years [39].

### 3.9. Predictive Value of a Nomogram in Patients with KIRC

**Figure 10:**
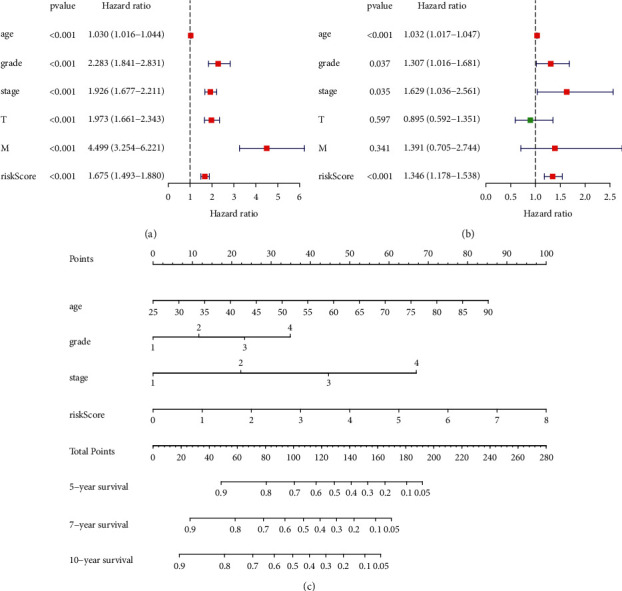
(a) Univariate Cox analysis. (b) Multivariate Cox analysis. (c) Nomogram of the model.

Univariate and multivariate Cox regression analyses were performed to analyze the relationship between RS and clinicopathological features of the KIRC patients. The results from univariate analyses showed that the clinicopathological features and RS correlated with the survival of the patients (Figure 10(a)). Moreover, the results from multivariate analyses demonstrated that the RS was an independent risk factor that correlated with the OS (Figure 10(b)). Lastly, we used a nomogram for predicting the outcome of patients with KIRC. There are a total of nine lines in the nomogram (Figure 10(c)), which from the second to the ninth lines represent age, grade, stage, RS, total points, and 5-, 7-, and 10-year survival, respectively. The patient scores were obtained and calculated together to generate the total scores from the second to the fifth row, corresponding to 5-, 7-, and 10-year survival (Figure 10(c)).

## 4. Discussion

The concept of angiogenesis refers originally to the formation of new blood vessels based on existing blood vessels in the process of organ development and wound healing, which form the basis for the existence of body functions. Angiogenesis also plays an extremely important role in the progression of malignant tumors (44). Hypermetabolism of malignant tumor cells induces the production of hypoxia-induced factors, which can lead to the secretion of angiogenic factors such as VEGF and PDGF (9). The well-known angiogenic factor VEGF family consists of six members (VEGFA–F) that play a critical role in angiogenesis through VEGFR3 and Neuropilin binding to the receptor VEGFR1 (45). In recent years, drug therapy targeting VEGF or VEGFR has become the main treatment for advanced renal cancer. RCC is the eighth most common carcinoma in USA, with an estimated incidence that may reach 74,000 in 2020 [40]. Although the response to molecularly targeted therapy is at the initial stage, most RCC patients ultimately suffer tumor progression. At present, molecularly targeted drug resistance is a huge challenge in achieving a better prognosis for advanced RCC [41]. Several studies have suggested that drug resistance results from the involvement of multiple factors such as tumor stem cells and the activation of other pathways [42]. PDGF is an angiogenic factor that can induce tumor angiogenesis and directly or indirectly promote the proliferation and migration of tumor cells. At present, the inhibition of tumor angiogenesis by inhibiting tyrosine-protein kinase activity and signal transduction of PDGF/PDGFR has become a hot topic in tumor therapy. However, the mechanism of PDGF and its related pathway genes in the occurrence and development of KIRC and its role in resistance to molecularly targeted drug therapy is still unclear. In addition, although great advances have been made in cancer genetics, we do not yet have a unified view of how to overcome tumors by combining genetic mutations, CNV, and other factors [43]. Gene CNV can affect the stability of gene expression, thus affecting gene transcription and expression, which correlates closely with the occurrence of many diseases, especially in malignant tumors. As an important cancer-promoting factor, the CNV of the genes related to the PDGF pathway are widely present in cancer. In our study, to clarify the role and mechanism of the PDGF gene and its pathway in the occurrence and development of renal cancer, genetic mutations of PDGF pathway genes were first detected in 32 types of cancer. Our results showed that CNV gain and CNV loss existed in most tumors. However, although only a few PDGF genes had CNV and SNV in KIRC, we found that there was a significant difference in the DSS, OS, and PFS in different CNV groups, suggesting that the CNV of PDGF genes could significantly affect the prognosis of KIRC patients. Subsequently, the alterations in the expressions of the PDGF-related genes were investigated and whether they had protective or risk roles in different tumors was confirmed. Our results suggest that most genes in the PDGF pathway have protective roles in KIRC, which was contradictory with previous results suggesting that the PDGF pathway exists as a cancer promoter. The potential causes of these contradictory results may be that undiscovered pathways exist and influence each other or that the tumors are heterogeneous. In addition, we also analyzed the relationship between the methylation of PDGF-related genes and patient prognosis by using GSCA. The results suggested that these PDGF gene methylations could significantly affect the prognosis of KIRC patients. Furthermore, we found that there was a positive correlation between CNV and mRNA expression, while there was a negative correlation between methylation and mRNA expression in most PDGF-related genes. Based on the above results, we could infer that the CNV and methylation of PDGF-related genes play a key role in KIRC progression by affecting the expression and translation of mRNA. Therefore, to further study the mechanism of the PDGF pathway in KIRC, the 539 KIRC samples obtained from TCGA were classified into three clusters based on PDGF scores and gene expression patterns. Our results demonstrate that patients in the PDGF-active cluster had higher OS rates than in the PDGF-inactive cluster, which indicated that the PDGF pathway gene has a protective role once again in KIRC. Unfortunately, the cause remains unclear.

Covalent modification of the histone occupies an important position in the epigenetic modification of the tumor, which includes phosphorylation, acetylation, and methylation. Among the histones with acetylation/deacetylation functions, HDACl of the HDAC family catalyzes the deacetylation of histones and maintains the balance between acetylation and deacetylation of histones, which is the main driving force of gene expression regulation [44]. As an important epigenetic regulator, the abnormal activity of histone deacetylases plays a significant role in the development and metastasis of tumors [45]. Studies have found that overexpressed HDAC1 can not only inhibit p53 and VHL but also induce the expression of HIF-1A and VEGF, which could increase tumor angiogenesis. In contrast, HDAC inhibitors could reduce angiogenesis and enhance tumor recognition by immune cells, which may contribute to their antitumor activity [46]. Therefore, further research on the interaction and mechanism between HDAC and the PDGF pathway genes in KIRC can provide a theoretical basis for the precise treatment of renal cancer in the future. In our study, there were significant differences in the expressions of the HDCA genes (except for HDAC6) in the three clusters. Moreover, HDAC1, HDAC5, HDAC7, HDAC8, HDAC10, and HDAC11 expressions were significantly higher in the PDGF inactive cluster than in the PDGF active cluster, indicating that they may be associated with the poor prognosis of the PDGF inactive cluster. Currently, the inhibition of HDAC has been used as a clinically proven cancer treatment strategy, and our research results can offer new directions for the future precision treatment of RCC. For instance, HDAC10 was significantly upregulated in the inactive group but was normal in the active group. Therefore, treatment with the HDAC10 inhibitor may be more beneficial to KIRC patients with the inactivation of PDGF pathway genes.

SIRT is a complex oncogene or tumor suppressor gene, which is involved in a variety of tumor-related molecular biological processes such as tumor-related metabolism, changes in the tumor microenvironment, and abnormal cell proliferation [31]. Our results showed that there were significant differences among the three clusters in the expression of SIRTs (except for SIRT3). The expressions of SIRT1 and SIRT5 in the inactive PDGF group were significantly lower than those in the active PDGF group, whereas SIRT2, SIRT4, SIRT6, and SIRT7 expressions in the inactive PDGF group were significantly higher than those in the active PDGF group. The ethanol extract of *P. scabiosifolia* can induce the death of 786-O cells via SIRT-1 and mTOR [32]. In summary, these results suggest that SIRT2 inhibitors may be more effective for patients who belong to the inactive PDGF group (C2).

Currently, a variety of molecularly targeted drugs with different mechanisms of action are widely used in clinical practice, such as sorafenib and sunitinib, which target the angiogenesis pathway [47, 48]. However, resistance to molecularly targeted drugs forms a huge challenge in the therapy of aRCC. The possible mechanisms of resistance to the antiangiogenic treatment of RCC are as follows: (1) upregulation of angiogenic factor expression [49], (2) changes in the tumor microenvironment [50], (3) the reversible transformation of epithelial cells into mesenchymal cells [51], (4) alternate upregulation of antivascular signaling pathways [52], and (5) alternation of VEGFR ligands [53]. In our study, the correlation between drug sensitivity and mRNA expressions of PDGF genes was investigated through the GDSC database and CTRP website. Results showed that all the drugs from GDSC and CTRP closely correlate with most PDFG pathway genes. Targeted drug therapy is an important treatment for advanced RCC, and the relationship between PDGF pathway genes and targeted drug therapy is worth studying. Based on the results of the relationship between molecularly targeted drugs and PDFG pathway genes, we may have a better understanding of the therapeutic effects of the drugs in the three clusters, which will help to establish precise treatments for KIRC in the future. For example, a patient with advanced KIRC and a high PDGF pathway gene expression and a high PDGF score may benefit from treatment with vorinostat or pazopanib. To solve the problem of targeted drug resistance and further improve the survival benefit of aRCC patients, it is necessary to further study the mechanism of action and drug resistance, deepen the understanding of the occurrence and development of RCC at the molecular level, and develop new targeted drugs.

Infiltrating immune cells play an important role in tumor metabolism and metastasis because they have an indispensable part in tumor immunology and microenvironment. At first, it was thought that these immune cells resulted from the host's immune response to the tumor. However, it is increasingly recognized that the body does not perceive tumor cells as foreign most of the time and that inflammatory/immune cell infiltration could promote tumor growth and metastasis [54, 55]. The distribution pattern of immune cells in the tumor microenvironment is closely related to the survival prognosis of patients, and the infiltrating pattern of immune cells can be used as the basis for treatment to improve the survival rate and long-term prognosis of patients [25]. As a subgroup of CD4+ helper T cells, Treg cells can significantly restrain the tumor immune response in the human body, thus promoting tumor progression [56]. In addition, mast cells are closely associated with tumor growth mediated by immune cells [54, 57]. Neutrophils have both protumor and antitumor functions depending on their state of differentiation [58]. In this study, we found that STAT1, STAT3, TIAM1, VAV1, SRC, RAC1, PLA2G4A, PAK1, NFKB1, and GRB2 had a significantly positive association with the infiltration of immune cells in KIRC. However, VAV2, WASL, RAF1, MAPK8, and ARFIP2 had a significantly negative association with the infiltration of immune cells. Furthermore, for most infiltration immune cells, especially Treg, neutrophils, and Type II IFN response, we found that there was a remarkable correlation with PDGF pathway genes and a significant difference, except for Th2 cells, Tfh, NK cells, and aDCs. Subsequently, the correlation of the PDGF score with immune cell infiltration was analyzed, and the results indicated that a positive correlation with the infiltration of type II IFN response cells and neutrophils existed. Our findings may be related to the complexity of the immune system and its interaction with the PDGF pathway. Improving the ability of the human immune system to recognize and kill tumor cells is an effective method of inhibiting tumor progression and metastasis. At present, tumor immunotherapy has become a first-line treatment for advanced renal cancer [59, 60]. Furthermore, a programmed cell death-1 inhibitor such as pembrolizumab combined with a tyrosine kinase inhibitor such as axitinib may result in a better prognosis than sunitinib for previously untreated advanced RCC patients [61]. However, how to change the types and roles of immune-infiltrating cells in the tumor microenvironment through PDGF pathway genes needs further study, which may provide a new direction and theoretical basis for the immunotherapy of RCC.

Finally, based on the expressions of PDGF pathway genes and the RS, LASSO regression analysis was used to select 11 genes for the construction of a risk model to predict the prognosis of KIRC patients. Based on their RS values, we classified patients with KIRC into high-risk and low-risk groups. The results showed that PDGF was closely related to the clinicopathological features of the KIRC patients. Meanwhile, a survival curve was drawn to show that the OS rate was significantly lower in the high-risk group than in the low-risk group. Subsequently, the results of the ROC curve analysis illustrated that the risk model had a good predictive value. Currently, prediction models of KIRC risk or survival based on different genes or pathways also exist. However, higher predictive accuracy was shown by our prognostic model compared with the signature constructed by Yu et al. in which the survival time of patients with KIRC was 1 year and 4 years [39]. The establishment of these models, combined with other methods, provides us with the possibility of effectively predicting the prognosis of patients with KIRC in the future.

Through this study, we not only clarified the mutation and expression of PDGF pathway genes in different cancers, indicating their key role in tumorigenesis, but also preliminarily demonstrated their protective role in KIRC, indicating that the PDGF pathway in KIRC was associated with classic tumor-related genes, histone modifications, and molecularly targeted drugs. Most importantly, the PDGF model constructed by us has a high predictive value for the prognosis of KIRC patients. In conclusion, the pathogenesis of KIRC is associated with an abnormally activated PDGF pathway, which may be a potential drug target in the treatment of KIRC. However, it is worth noting that this study was conducted using bioinformatics analysis, so relevant research results need to be further verified through experimental or clinical practice in future studies.

## Data Availability

The data used to support the findings of this study are available from the corresponding authors upon request.

## Abbreviations

KIRC: Kidney renal clear cell carcinoma
RCC: Renal cell carcinoma
THCA: Thyroid carcinoma
THYM: Thymoma
PRAD: Prostate adenocarcinoma
UCEC: Uterine corpus endometrial carcinoma
SKCM: Skin cutaneous melanoma
COAD: Colon adenocarcinoma
TCGA: The Cancer Genome Atlas
GDSC: Genomics of Drug Sensitivity in Cancer
HPA: The Human Protein Atlas
LASSO: Least absolute shrinkage and selection operator
GSEA: Gene set enrichment analysis
CMap: Connectivity map
CNV: Copy number variation
SNV: Single-nucleotide variation
VEGF: Vascular endothelial growth factor
TME: The tumor microenvironment
HRAS: HRas protooncogene
MYC: Myelocytomatosis oncogene
VEGFA: Vascular endothelial growth factor A
VHL: Von Hippel-Lindau
PTEN: Phosphatase and tensin homolog
EGFR: Epidermal growth factor receptor
STAT3: Signal transducer and activator of transcription 3
HDAC: Zinc-dependent histone deacetylases
SIRT: Sirtuins.
